# MicroRNA-155 regulates tumor myeloid-derived suppressive cells

**DOI:** 10.18632/oncoscience.269

**Published:** 2015-11-19

**Authors:** Siqi Chen, Yi Zhang, Bin Zhang

**Affiliations:** Biotherapy Center, The First Affiliated Hospital of Zhengzhou University, Zhengzhou, Henan, China; Robert H. Lurie Comprehensive Cancer Center, Department of Medicine-Division of Hematology/Oncology, Northwestern University Feinberg School of Medicine, Chicago, IL, USA

**Keywords:** microRNA, MDSC, tumor microenvironment, immunosuppression

MicroRNA is small non-coding RNA and can lead to translational repression or target degradation by base-pairing with complementary sequences of mRNA molecules. MicroRNA-155 (miR-155), one of the most studied microRNA, is the first one to be reported as oncogenic [1]. miR-155 is over expressed in a long list of both hematological and solid tumors and is of paramount importance in cancer diagnosis and prognosis. However, how miR-155 particularly in host immune system regulates the tumor progression remains poorly understood. Our study underscores a contextual role of miR-155 in regulating tumor growth and tumor immunity via distinct immune subsets within tumors [2]. We conclude that the balance of different effects between those immune cell populations, which are regulated by miR-155, appears to determine whether miR-155 promotes or inhibits tumor growth [2].

We demonstrated that host miR-155 deficiency promoted antitumor T cell immunity in multiple transplanted tumor models. Further analysis of immune cell compartments revealed that miR-155 was required for the accumulation and suppressive function of myeloid-derived suppressive cells (MDSC) in the tumor microenvironment. Apart from the direct modulation on MDSC, miR-155 was also required for the MDSC-mediated CD4^+^Foxp3^+^ regulatory T cells (Treg) induction. On the other hand, miR-155 deficiency hampered the antitumor responses of both dendritic cells and T cells. Therefore, it appears that in our tumor models, miR-155 mediated a dominant immunosuppressive effect by MDSC, leading to the enhanced overall antitumor immunity in miR-155 deficient hosts.

Reduced colon inflammation and decreased colorectal carcinogenesis were also found in miR-155 deficient mice when azoxymethane (AOM) and dextran sodium sulphate (DSS) were combined to induce colon lesions. Furthermore, miR-155 was upregulated in MDSC either from tumor-bearing hosts or generated from bone marrow progenitors by GM-CSF and IL-6. These results support the notion that miR-155 is a prototypical microRNA bridging inflammation and cancer development [3]. Although miR-155 may regulate tumor growth in an intrinsic manner, it is likely that inflammation promotes the accumulation of functional MDSC by increased miR-155 that dampens the immune surveillance and antitumor immunity, thereby facilitating tumor growth.

**Figure 1:**
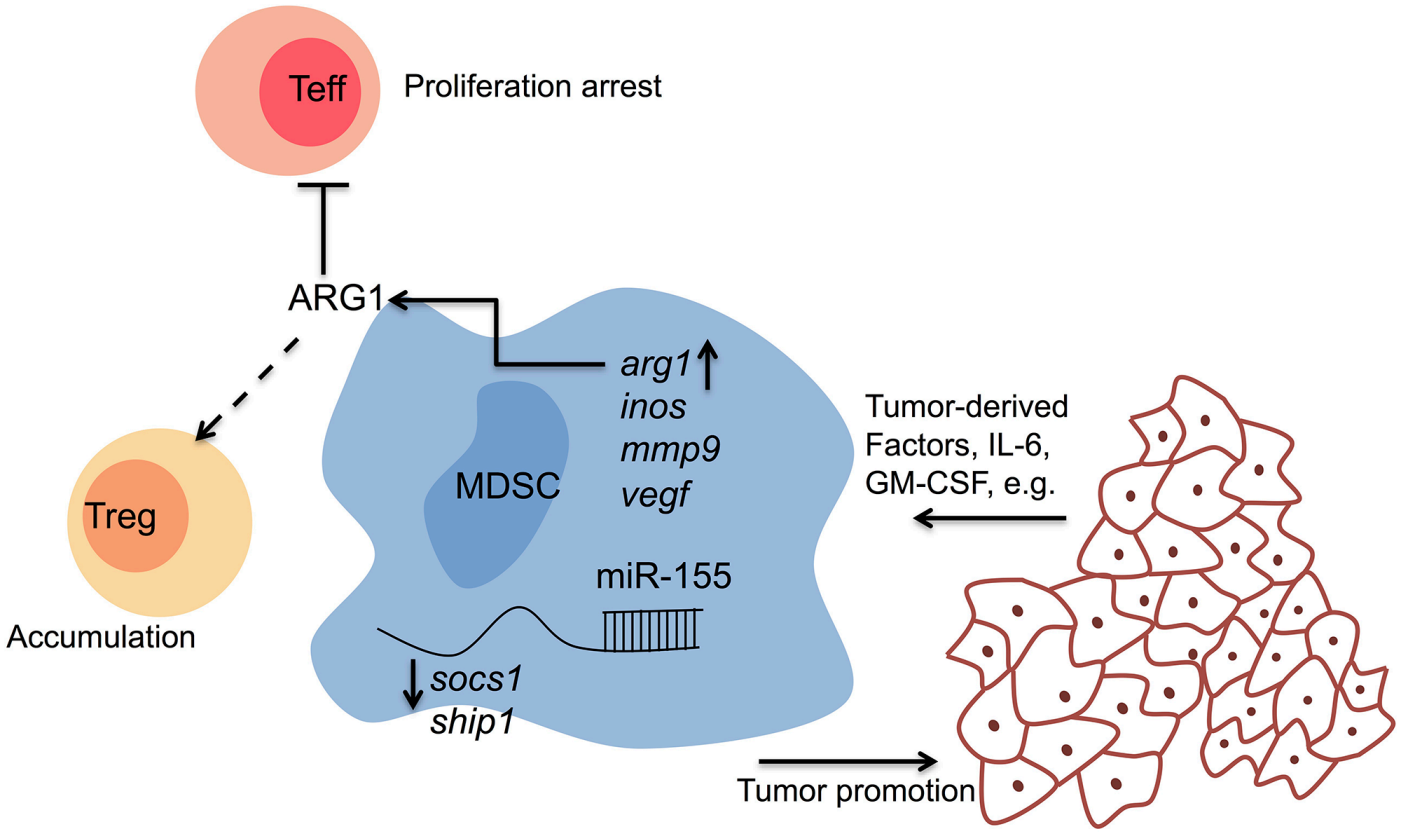
miR-155 regulates tumor MDSC Tumor-derived factors such as GM-CSF and IL-6 upregulate the expression of miR-155, which could be a key player in balancing anti-and pro-tumor immune components within the tumor. In our given tumor model system, miR-155 promotes tumor growth in an MDSC-dependent manner. It appears that both SHIP-1 and SOCS1 are important target genes of miR-155 during functional MDSC generation. Further, miR-155 may modulate arginase-dependent suppressive activity of MDSC. Apart from the direct modulation on MDSC, miR-155 is required for the MDSC-mediated Treg induction.

To identify the molecular mechanisms by which miR-155 regulates MDSC (Figure 1), we found that miR155 retained the suppressive activity of MDSCs through inhibiting SOCS1. Moreover, inverse correlations between miR-155 expression and SHIP-1/SOCS1 expression were established in MDSC. As SHIP-1 was recently reported as a target of miR155 specifically in MDSC expansion [4], these results suggest both SHIP-1 and SOCS1 as target genes of miR-155 during functional MDSC generation. SOCS1 also restricted arginase I activity [5], which otherwise would limit the efficiency of MDSC proinflammatory responses. Indeed, we showed that miR-155^−/−^ MDSC has a lower level of arginase activity than WT counterparts, and inhibition of arginase-I with specific inhibitors completely abrogated the suppressive activity of WT MDSC and did not affect the miR-155^−/−^ MDSC. Our data indicate that miR-155 may modulate arginase-dependent suppressive function of MDSC via targeting SOCS1.

More interestingly, we observed the decreased production of MMP-9 and VEGF from miR-155^−/−^ MDSC, which would presumably limit the tumor angiogenesis. Given a contribution of miR-155 expression by cancer cells to tumor angiogenesis [6], further studies will determine whether miR-155 regulates tumor angiogenesis through both cancer cells and MDSC within tumors.

It is notable that our results on host miR155 deficiency and tumor growth differ from other recent studies [7, 8]. Differences in the tumor cell lines used that could change the accumulation of individual immune cell subsets in the tumor microenvironment may explain this discrepancy. The extent and modulation of major immune populations could vary in different tumor types and/or tumor stages. Thus, increased miR-155 could be a key player in balancing anti-and pro-tumor immune components within the tumor. In our given tumor model system, we provide clear evidence that miR-155 promotes tumor growth in an MDSC-dependent manner, as manifested via both “depletion” and “transfer” strategy *in vivo*.

Taken together, our study highlights the essence of evaluating the intrinsic role of miR-155 carefully in distinct immune cell subsets, where miR-155 could be either protective or deleterious to antitumor immunity. In this regard, it would be safe and important to develop the anti-miR-155 cancer therapy in cell-specific manner. It is becoming evident that miR-155 functions as “OncomiR” in concert with “ImmunomiR” in orchestrating cancer growth and progression.
